# An external pilot cluster randomised controlled trial of a theory-based intervention to improve appropriate polypharmacy in older people in primary care (PolyPrime)

**DOI:** 10.1186/s40814-022-01161-6

**Published:** 2022-09-10

**Authors:** Audrey Rankin, Ashleigh Gorman, Judith Cole, Cathal A. Cadogan, Heather E. Barry, Ashley Agus, Danielle Logan, Cliona McDowell, Gerard J. Molloy, Cristín Ryan, Claire Leathem, Marina Maxwell, Connie Brennan, Gerard J. Gormley, Alan Ferrett, Pat McCarthy, Tom Fahey, Carmel M. Hughes, on behalf of the PolyPrime team, Lynn Murphy, Gavin Kennedy, Catherine Adams, Laurie Martin, Joanne Thompson, Sorcha Toase, Carys Boyd, Rachael McQuillan, Máire O’Dwyer

**Affiliations:** 1 School of Pharmacy, Queen’s University Belfast, Belfast, BT9 7BL UK; 2 School of Pharmacy and Pharmaceutical Sciences, Trinity College Dublin, Dublin, Ireland; 3 Northern Ireland Clinical Trials Unit, Belfast, UK; 4 School of Psychology, National University of Ireland, Galway, Ireland; 5 Northern Ireland Clinical Research Network (Primary Care), Belfast, UK; 6 School of Medicine, Dentistry and Biomedical Sciences, Queen’s University Belfast, Belfast, UK; 7 Public Involvement Enhancing Research,, Belfast, Northern Ireland, UK; 8 Donegal Volunteer Centre, Donegal, Ireland; 9 Department of General Practice, Royal College of Surgeons in Ireland, Dublin, Ireland

**Keywords:** Polypharmacy, Behaviour change, Primary care, General practice, Complex intervention, Pilot study, Process evaluation, Older people, Prescribing

## Abstract

**Background:**

For older populations with multimorbidity, polypharmacy (use of multiple medications) is a standard practice. PolyPrime is a theory-based intervention developed to improve appropriate polypharmacy in older people in primary care. This pilot study aims to assess the feasibility of the PolyPrime intervention in primary care in Northern Ireland (NI) and the Republic of Ireland (ROI).

**Methods:**

This external pilot cluster randomised controlled trial (cRCT) aimed to recruit 12 general practitioner (GP) practices (six in NI; six in the ROI counties that border NI) and ten older patients receiving polypharmacy (≥ 4 medications) per GP practice (*n* = 120). Practices allocated to the intervention arm watched an online video and scheduled medication reviews with patients on two occasions. We assessed the feasibility of collecting GP record (medication appropriateness, health service use) and patient self-reported data [health-related quality of life (HRQoL), health service use)] at baseline, 6 and 9 months. HRQoL was measured using the EuroQol-5 dimension-5 level questionnaire (EQ-5D-5L) and medication-related burden quality-of-life (MRB-QoL) tool. An embedded process evaluation and health economics analysis were also undertaken. Pre-specified progression criteria were used to determine whether to proceed to a definitive cRCT.

**Results:**

Twelve GP practices were recruited and randomised. Three GP practices withdrew from the study due to COVID-related factors. Sixty-eight patients were recruited, with 47 (69.1%) being retained until the end of the study. GP record data were available for 47 patients for medication appropriateness analysis at 9 months. EQ-5D-5L and MRB-QoL data were available for 46 and 41 patients, respectively, at 9 months. GP record and patient self-reported health service use data were available for 47 patients at 9 months. Health service use was comparable in terms of overall cost estimated from GP record versus patient self-reported data. The intervention was successfully delivered as intended; it was acceptable to GPs, practice staff, and patients; and potential mechanisms of action have been identified. All five progression criteria were met (two ‘Go’, three ‘Amend’).

**Conclusion:**

Despite challenges faced during the COVID-19 pandemic, this study has demonstrated that it may be feasible to conduct an intervention to improve appropriate polypharmacy in older people in primary care across two healthcare jurisdictions.

**Trial registration:**

ISRCTN, ISRCTN41009897. Registered 19 November 2019. Clinicaltrials.gov, NCT04181879. Registered 02 December 2019.

**Supplementary Information:**

The online version contains supplementary material available at 10.1186/s40814-022-01161-6.

## 1) What uncertainties existed regarding the feasibility?

- There were uncertainties regarding the feasibility of sampling and recruitment, intervention delivery and outcome data collection procedures across the two healthcare jurisdictions (NI and the ROI).

## 2) What are the key feasibility findings?

- This pilot study has confirmed that despite the challenges faced due to the COVID-19 pandemic, it is feasible to recruit GP practices and deliver the PolyPrime intervention in primary care across two healthcare jurisdictions.
- It is feasible to collect GP record and patient self-reported data at baseline, 6 and 9 months, with high return rates and completeness of data.
- It is feasible to collect the GP record data required to apply the Screening Tool of Older People’s potentially inappropriate Prescriptions (STOPP)/Screening Tool to Alert doctors to Right Treatment (START) criteria in order to assess medication appropriateness using the robust case report form developed.

## 3) What are the implications of the feasibility findings for the design of the main study?

- The findings from this study can be used to inform changes to study procedures in any subsequent trial including the data collection timepoints used and selection of outcome measures (i.e. medication appropriateness and HRQoL).
- Further work is warranted to establish the effectiveness of the patient recruitment strategies in place and the ability to retain patients for the duration of the study.
- Further work is also needed to investigate potential adaptations to the delivery of the intervention package in terms of the workforce in primary care (i.e. the role of practice-based pharmacists) and the use of telephone and video consultations.

## Background

Polypharmacy, defined as the ongoing use of multiple medicines (≥ 5 medicines), is a standard practice for older adults (conventionally defined as ≥ 65 years) with multimorbidity [1]. Studies from the United Kingdom (UK) and the Republic of Ireland (ROI) have estimated that between 20 and 30% of older adults are currently dispensed five or more medications [2, 3]. Medication use and safety have also become a global public health issue in many countries in line with the World Health Organizations (WHO) Global Patient Safety Challenge: Medication Without Harm, which highlights polypharmacy as a major priority [4, 5].

The challenge faced by general practitioners (GPs) in primary care is achieving a balance between many drugs (appropriate polypharmacy) and too many drugs (inappropriate polypharmacy) [1]. Prescribing has been described as a complex clinical behaviour, encompassing several specific actions [6] and requiring a range of skills (e.g. assessment, knowledge, judgement) [7]. Therefore, the promotion of appropriate polypharmacy requires interventions which encourage a change in prescribing behaviour. Several interventions have been developed to address appropriate polypharmacy in older people including medication reviews, using risk screening tools and computerised clinical decision support systems aimed at prescribers [8]. However, it remains unclear whether the interventions result in clinically significant improvements in hospital admissions, medication-related problems including adherence, and patients’ overall quality of life [8].

Previous work conducted by members of the research team has developed an intervention to address the challenge of achieving appropriate polypharmacy in primary care which is described in detail elsewhere [9–11]. The intervention package consists of four components: a short online video, a patient recall process, weekly practice meetings and prompts delivered to the GPs [10, 11]. An initial feasibility study conducted in two GP practices in Northern Ireland (NI) found that the intervention was acceptable to GPs and patients [11] and highlighted several areas for refinement.

The PolyPrime project follows on from this programme of work to undertake a larger study in a cross-border setting (NI and the border region of the ROI: Cavan, Donegal, Leitrim, Louth, Monaghan and Sligo). Minor refinements were made to the intervention (following interviews with ROI GPs) before commencement of the main pilot study [12, 13]. The current paper reports on the external pilot cluster randomised controlled trial (cRCT), with an embedded process evaluation and health economic analysis. The primary aim was to assess the feasibility of a definitive cRCT of the effectiveness and cost-effectiveness of the PolyPrime intervention in primary care in NI and the ROI. The objectives of the study were as follows:

1. Test approaches to sampling, recruitment and retention of GP practices and patients.
2. Test the feasibility of using medication appropriateness as the primary outcome in a future cRCT.
3. Identify the resources used in the set-up and delivery of the intervention and their associated costs.
4. Assess the feasibility of a future cost-effectiveness analysis.
5. Further validate the medication-related burden quality-of-life (MRB-QoL) tool.
6. Obtain estimates of effect size between groups, cluster size and intraclass correlation coefficients to inform the sample size calculation for a full cRCT.
7. Identify the intervention’s likely mechanism of action.
8. Assess if the intervention is delivered and received as intended (intervention fidelity).
9. Assess if the intervention is acceptable to GPs, practice staff and patients.

Pre-specified progression criteria (outlined below) based on recruitment and retention of GPs and patients, and completeness of outcome data, were used to determine whether to proceed to a definitive cRCT or if further modifications are warranted.

## Methods

### Study design and participants

The methods for the PolyPrime study including a detailed overview of the PolyPrime intervention and the recruitment procedures for GP practices and patients have been described in detail elsewhere [13]. Due to COVID-19 restrictions in place in both NI and the ROI, the PolyPrime study was suspended between March and July 2020. A number of changes were made to the original study including the use of either telephone or online/video consultations and the data collection timepoints (see ‘Intervention overview’ and ‘Outcome data collection’ sections).

Briefly, PolyPrime is an external pilot cRCT conducted in GP practices in two healthcare systems (NI and the six counties in the ROI that border NI). The study aimed to recruit 12 GP practices across NI and the ROI border counties, with one GP practice per county. Practices were recruited in two stages: expression of interest letters followed by telephone calls conducted by research nurses from the Northern Ireland Clinical Research Network (NICRN – Primary Care) and Trinity College Dublin (TCD). GP practices were eligible if they provided written informed consent and research governance sign off, had a stable Internet service to access the video and were not currently participating in other studies related to medicines management in older people. Potentially eligible patients were identified via GP records, with the aim of recruiting up to 10 patients per practice. Patients were eligible if they were ≥ 70 years old; receiving four or more regular medicines (i.e. prescribed for more than 3 months); not cognitively impaired; did not have a terminal illness; were resident in the community; in receipt of a valid general medical services (GMS) card in the RoI, or for NI patients; registered for National Health Service (NHS) primary care services; and registered with and/or regularly attending the practice for a minimum of 12 months. Eligible patients were posted information packs, including an invitation letter along with an information sheet, consent form and baseline questionnaires (see ‘Outcome data collection’ section).

When the study recommenced (July 2020), a number of GP practices and patients had already provided written informed consent. A process of re-engaging with GP practices and patients was undertaken to ascertain if they were still willing to participate. Patients were contacted by letter, outlining the changes which had been made to the study and giving them the option of remaining in or withdrawing from the study. In addition, patients already recruited from a GP practice which withdrew after baseline data collection, were notified about the practice withdrawal and asked if they were still willing to participate by returning self-report questionnaires (see ‘Outcome data collection’ section).

### Randomisation and blinding

Recruited practices were allocated to intervention (*n* = 6) or control groups (*n* = 6) according to a randomisation schedule that was generated by an NICTU statistician (using nQuery Advisor®) and saved in a restricted section of the trial master file. Practices were randomised on a 1:1 allocation ratio stratified by country (i.e. NI or ROI). It was not possible to blind GPs or patients due to the nature of the intervention. In order to reduce detection bias, the primary outcome measure (medication appropriateness) was assessed by blinded pharmacists in the team (CH, HB, CC, CR).

### Intervention overview

The PolyPrime intervention package consisted of an online video [incorporating behaviour change technique (BCT): ‘modelling or demonstrating of behaviour’ and ‘salience of consequences’ [14]] and a patient recall process. Intervention arm GPs received unique log-in details to the online video, where they also had access to supplementary material (i.e. tools used to support medication reviews including the Screening Tool of Older People’s potentially inappropriate Prescriptions (STOPP)/Screening Tool to Alert doctors to Right Treatment (START) criteria and NO TEARS tool [15, 16], in addition to where to go for further information [i.e. National Institute for Health and Care Excellence (NICE) guidance for medicines optimisation [17]). GPs were then instructed to complete medication reviews with the recruited patients on two occasions (an initial medication review and a 6-month follow-up medication review). Due to COVID-19 restrictions during the intervention delivery phase, appointments took place face to face, via the telephone or video call. Two complementary intervention components were also included, whereby GPs were asked to have weekly practice meetings to discuss how and when patient appointments would take place (BCT: action planning [14]), and practice staff was instructed to prompt GPs to conduct a medication review when patients presented for a scheduled appointment (BCT: prompts/cues [14]).

Control arm GPs continued to deliver usual care to recruited patients during the study period. At the time of GP practice recruitment, no structured chronic disease management programme had been embedded into primary care in the ROI. In January 2020, the Health Service Executive (HSE) in the ROI launched a Chronic Disease Management Programme involving reviews for patients ≥ 70 years old with asthma, type 2 diabetes, chronic obstructive pulmonary disease (COPD) or cardiovascular disease [18]. All GP practices in the study were asked to give an overview of their current prescribing practices (usual care) for older patients receiving polypharmacy, including whether medication reviews were being routinely conducted and by whom (i.e. GPs, pharmacists).

### Sample size

As previously reported, formal sample size calculation was not undertaken [13]. However, based on previous research experience, a target of 10 patients per GP practice (i.e. a maximum of 120 patients in total across 12 GP practices) was deemed appropriate to provide sufficient data to meet the objectives of this pilot study.

### Outcome data collection

Outcome data (patient self-report and GP record) were collected at baseline, 6-month and 9-month (previously 12 months) post-intervention for intervention patients (i.e. after the patients’ initial medication review) [13]. Follow-up timepoints for the control arm were based on the average length of time from the completion of baseline data collection to 6- and 9-month post-initial medication review in the intervention arm.

### Primary outcome

The primary outcome was medication appropriateness measured using STOPP/START criteria [16]. STOPP/START consists of a set of 114 explicit criteria which help clinicians detect common instances of potential inappropriate prescribing (PIP) which encompasses potentially inappropriate medicines (PIMs) and potential prescribing omissions (PPOs). A case report form (CRF) was developed to collect patient data [including medical history, clinical conditions, biochemical data (i.e. test results) and prescribed medications] from GP files by the research nurses. Data collected were used to assess prescribing appropriateness by four blinded pharmacists on the research team.

### Secondary outcomes

Secondary outcomes included health service use (i.e. hospitalisations) (see ‘Health service use and associated costs’ section) and health-related quality of life (HRQoL) (see ‘Health outcomes’ section).

### Process evaluation

A detailed overview of the process evaluation approach has been described elsewhere [19]. Briefly, a mixed methods process evaluation ran in parallel to and following the completion of the intervention to investigate the acceptability of, fidelity to and the likely mechanisms of action of the PolyPrime intervention. Quantitative data were collected using study-specific data collection forms completed by practice staff and a feedback questionnaire completed by patients from intervention arm practices. Qualitative data were collected through semi-structured interviews with GPs and practice staff and audio recordings of medication review appointments from the intervention arm practices.

### Statistical analysis

Analysis was conducted STATA®/IC version 15.1 (StataCorp). For descriptive statistics, the mean and standard deviation (SD) were calculated for normally distributed continuous data, counts and percentages presented for categorical data, and the median and interquartile range (IQR) were estimated for skewed or ordinal data. For the medication appropriateness data, the proportion of patients with at least one instance of PIP, PIM and/or PPOs, at baseline, 6 months and 9 months, was calculated. The percentage point difference (95% CI) and intraclass correlation coefficient (ICC) were provided at 6 months and 9 months. The 95% confidence intervals were based on the default exact binomial method used for proportions in STATA, and the ICCs were estimated from mixed effects models with site as a random effect. The individual criteria involved in the identification of the instances of PIP, PIM and PPO at baseline, 6 months and 9 months, were also identified. A number of sample size calculations for a future definitive cRCT were undertaken based on the primary outcome (i.e. the proportion of patients with at least one instance of PIP) using Epitools [20]. Sample sizes were inflated for the design effect based on varying ICCs and cluster sizes.

### Health economic analysis

#### Intervention-related resource use and costs

Intervention-related resource use was categorised according to the stage at which they were used in the research process: planning and preparation for delivery (stage 1) and intervention delivery (stage 2). Intervention arm GPs were asked to complete a study-specific data collection form on which they estimated their time spent preparing and planning for the medication reviews and their time spent undertaking the medication reviews and performing any post-review activities. The time spent viewing the online video was based on the assumption that each intervention GP (*n* = 5) watched the video for its entire duration once (13 min, 14 s), divided by the number of patients recruited to the intervention arm. Costs were estimated by multiplying the GP time input by the cost per minute of £4.23 (based on an average 9.22 min consultation recorded by GPs on study-specific data collection forms and GP surgery consultation costs [21]), which was the same for both NI and ROI.

#### Health service use and associated costs (secondary outcome: health service use)

Health service use was obtained from GP records and using a bespoke patient self-reported questionnaire. Data were collected at baseline for the previous 6 months, at 6-month post-initial medication review (or the equivalent in the control arm) for the previous 6 months and at 9-month post-initial medication review (or the equivalent in the control arm) for the previous 3 months.

Health service use costs for both methods were calculated by attaching country-specific unit costs to the resource use data from the Department of Health National Schedule of Reference Costs [22], Unit Cost of Health and Social Care [21], Unit costs for non-acute care in Ireland 2016-2019 [23] and the Healthcare Pricing Office [24]. If a unit cost for a service was available in one country but not the other, then the same unit cost was used for both countries. All costs were expressed in 2019/2020 Great British pounds (GBP, £). ROI costs (in Euro, €) were converted to GBP using the 2019 purchasing power parities [25].

COVID-19 impacted on the health economic data collected over the course of the study, and due to delays in being able to access practices, there were discrepancies in the timepoints at which baseline health service use data (GP record versus patient self-report) were collected, meaning these data were no longer comparable. These data, therefore, have not been reported.

For each patient, the number of units (e.g. packs) of each drug used over the study period was estimated using information on the dose, frequency and prescription start and finish dates collected by the research nurses from the GP records. A maximum unit of 1 was assumed for each time period (i.e. baseline to 6-month follow-up, 6- to 9-month follow-up) when a medication that was taken as needed (PRN, pro re nata), when the frequency information was missing or when the medication was a gel, cream, inhaler or drop [26].

#### Health outcomes (secondary outcome: HRQoL)

HRQoL was measured using the EQ-5D-5L questionnaire (UK and Ireland versions) and the medicine-related burden quality-of-life (MRB-QoL) tool. The EQ-5D-5L contains two sections: five statements about mobility, self-care, usual activities, pain/discomfort and anxiety/depression and a visual analogue scale (EQ-VAS) [27]. The MRB-QoL tool is a validated measure of the burden of medicines on functioning and wellbeing. The MRB-QoL contains 31 items categorised under five subscales: ‘Routine and Regimen Complexity’ (11 items), ‘Psychological Burden’ (six items), ‘Functional and Role Limitation’ (seven items), ‘Therapeutic Relationship’ (three items) and ‘Social Burden’ (four items) [28].

Responses on the EQ-5D-5L were converted to utility scores using the crosswalk value set [29] as recommended by NICE in their guide to technology appraisal [30]. Responses on the MRB-QoL were converted to a total score out of 100 using the formula provided by the tool developers [28]. The EQ-5D-5L utility score could range from −0.594 and 1.00, where 0 reflects the worst possible health state and 1.00 the best possible health state. Minus scores are health states that were worse than death. The MRB-QoL score could range from 0 to 100, where 0 indicated no medication-related burden and thus best possible medication-related quality of life. In contrast, 100 indicates the highest level of burden and thus the worst possible medication-related quality of life. If a response was missing on an item in either instrument, then the overall score could not be calculated for that patient, and a missing score was recorded. The rates of missing data and ceiling and floor effects for each instrument were summarised at each timepoint and intervention group.

### Process evaluation analysis

A detailed overview of the quantitative and qualitative data analysis methods used in the process evaluation has been previously described [19]. Briefly, quantitative analysis was conducted using SPSS (version 26.0). For descriptive statistics, the mean and standard deviation (SD) were calculated for normally distributed continuous data, counts and percentages presented for categorical data, and the median and IQR were estimated for skewed or ordinal data. For the qualitative data, semi-structured interviews with GPs and practice staff and audio recordings of medication review appointments were transcribed and analysed using the framework method. Coding of the feedback interviews adopted a deductive approach using the Theoretical Framework of Acceptability (TFA) constructs [31] (used to frame questions relating to the overall acceptability of the intervention in GP and practice staff interviews [20]) and an inductive approach, in which emerging themes were identified. Coding of the medication review appointment transcripts adopted a deductive approach using the BCT taxonomy [14]. In addition, established methods [32] were used to link BCTs to theoretically defined mechanisms of action involved in the PolyPrime intervention.

### Progression criteria

Pre-specified progression criteria [13] were applied to determine whether to proceed to a definitive cRCT of the PolyPrime intervention or if further modifications were warranted. The cut-off points were based on work published by Borelli et al. [33], whereby when ≥ 80% of the target is met, the criteria meet the ‘Go’ thresholds, when 50% of the target is met, the criteria meet the ‘Amend’ thresholds or when < 50% of the target is met, the criteria meet the ‘stop’ thresholds. The criteria were based on GP practice recruitment and retention, patient recruitment and retention and completeness of the outcome data. A final decision was made on whether to proceed to a definitive cRCT through discussion with the Trial Steering Committee (TSC; [13]).

### Serious adverse events (SAEs)

GP practices were asked to complete a serious adverse event (SAE) reporting form monthly. Any events which took place in the intervention arm were clinically assessed by two academic GPs on the research team. If any SAEs linked to the PolyPrime intervention were deemed to be a suspected unexpected serious adverse reaction (SUSAR), these were to be reported to the TSC and sponsor for follow-up [13].

### Ethical approval, reporting and patient/public involvement

The study was granted ethical approval by the north of Scotland (REC reference: 19/NS/0100) and the Irish College of General Practitioners Research Ethics Committees (RECs); the study protocols were published in advance [13, 19]. This study has been reported in line with the Consolidated Standards of Reporting Trials (CONSORT) extension for reports of randomised pilot and feasibility studies [34]. A completed CONSORT checklist can be found in Additional file 1. Two patient and public involvement (PPI) representatives were members of the Project Management Group that provided advice to the research team during the study and reviewed all patient facing trial documentation.

## Results

### GP practice sampling, recruitment and retention

Twelve GP practices were recruited over a 6-month period (end of July 2019 to early January 2021). Expression of interest letters was posted to 160 GP practices [85 in NI, 75 in the ROI], and 9 reply slips expressing interest were returned [4 in NI, 5 in the ROI; overall response rate (letter) = 5%]. From those expressing interest, 6 practices were recruited [1 in NI, 5 in the ROI; overall recruitment rate (letter) = 4%]. Thereafter, research nurses contacted GP practices (*n* = 41) via telephone until the final six practices were recruited.

**Fig. 1:**
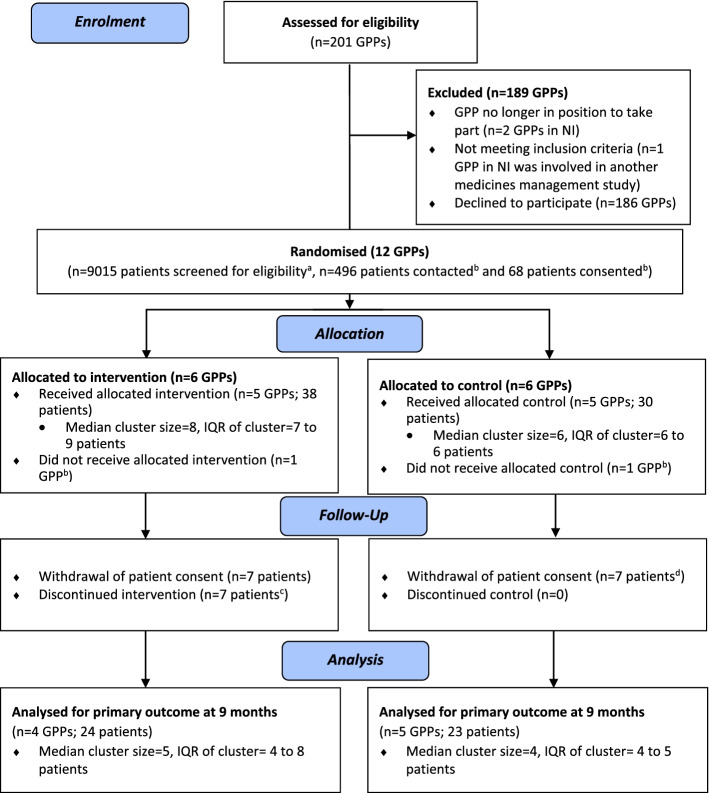
CONSORT flow diagram for the PolyPrime study. GPP, general practitioner practice; IQR, interquartile range. ^a^The number of patients screened for eligibility relates to 8 GPPs as information was not available for 4 GPPs. ^b^The number of patients contacted and consented relates to 10 GPPs as two GPPs withdrew from the study after randomisation but before baseline data collection and before any details on patient numbers could be obtained. ^c^One intervention arm GPP withdrew from the study, and patients did not receive any medication reviews; however, 3 patients were followed up for the patient-reported outcome questionnaires. ^d^An additional patient withdrew consent from study, but the primary outcome data were collected prior to withdrawal

When the study recommenced in July 2020, one GP practice withdrew due to COVID-related factors, and the study was unable to proceed at another practice as the NICRN-PC staff was seconded to COVID-19 studies in March 2020. One further GP practice in ROI withdrew from the study after baseline data collection due to workload pressures caused by COVID-19. A CONSORT diagram showing GP practice recruitment and retention is presented in Fig. 1.

Thirteen GPs were recruited from the 12 GP practices initially recruited. Baseline characteristics were collected from the 10 GP practices (5 in NI, 5 in the ROI) which were retained after study recommencement (see Table 1). Practices ranged from small single-handed practices to larger practices with six or seven GPs. The mean patient list size was comparable across the two study arms, but list sizes were larger in NI practices compared to those in ROI. Medication reviews were being conducted at six GP practices (two interventions and four controls) by practice staff (GPs, pharmacists or nurses) on a regular basis as part of standard practice (i.e. irrespective of their involvement in the PolyPrime study).

**Table 1:** GP practices’ baseline characteristics

	Intervention ***n*** = 5 GPPs	Control ***n*** = 5 GPPs
**Number of GP practices**		
NI *n* (%)	2 (40.0%)	3 (60.0%)
ROI *n* (%)	3 (60.0%)	2 (40.0%)
**Number of GPs within GP practices**		
Overall mean (SD)	4.0 (2.8)	4.4 (2.07)
NI mean (SD)	7.0 (0.0)	5.3 (1.2)
ROI mean (SD)	2.0 (1.0)	3.0 (2.8)
**Number of other staff members per practice**^a^		
Overall mean (SD)	11.8 (6.7)	10.6 (5.7)
GPP missing *n* (%)	1 (20.0)	0 (0.0)
**Patient list size**		
Overall mean (SD)	5168.4 (5005.6)	5738.4 (3259.9)
NI mean (SD)	10496.0 (377.6)	6714.0 (2440.0)
ROI mean (SD)	1616.7 (1654.0)	4275.0 (4843.7)
**Total years practising for all GPs**^b^		
Overall mean (SD)	15.3 (10.3)	31.20 (5.8)
GPP missing *n* (%)	1 (20.0)	0 (0.0)
**Number of GP practices using medication reviews**		
Overall *n* (%)		
Yes *n* (%)	2 (40.0)	4 (80.0)
No *n* (%)	2 (40.0)	0 (0.0)
GPP missing *n* (%)	1 (20.0)	1 (20.0)

*GPP* general practitioner practice. Missing represents the number of GP practices where data were not available

^a^Includes both part-time and full-time staff who are either administrative and support staff, nurses, pharmacists or other. ^b^Based on those GPs who were involved in the study (i.e. 11 GPs; one control practice had two participating GPs)

### Patient screening, recruitment and retention

Patient screening and recruitment took place in 10 GP practices between January 2020 to mid-March 2020 and mid-September 2020 to mid-November 2020. Invitation packs were posted to 496 patients (300 in NI, 196 in the ROI) from 10 GP practices, which resulted in 68 patients being recruited (32 in NI, 36 in the ROI). Of these patients, 28 (41.2%) were female [18 (47.4%) in the intervention arm, 10 (33.3%) in the control arm]. GP practices recruited between 4 and 10 patients each (median: 6.5), with one practice recruiting 10 patients and three practices each recruiting 8 patients.

When the study was restarted in July 2020, 12 patients (2 in NI, 10 in the ROI; 7 in the intervention arm, 5 in the control arm) withdrew consent before baseline data collection; data were collected from the 56 patients who were retained (see Table 2). The mean age of these patients was 76.4 (*SD*: ±4.4), and they were prescribed a mean of 6.7 (*SD*: 2.0) medications. Three-quarters had medical conditions which were cardiovascular in nature.

**Table 2:** Baseline patient characteristics at trial entry

	Intervention ***n*** = 31	Control ***n*** = 25	Total ***n*** = 56
**Mean age** (SD)	76.5 (3.7)	76.4 (5.2)	76.4 (4.4)
**Number of prescribed medications**^a^ mean (SD)	6.8 (1.9)	6.6 (2.1)	6.7 (2.0)
**Medical conditions (current or previous)**^b^*n* (%)			
Cardiovascular	19 (61.3)	23 (92.0)	42 (75.0)
Central nervous	2 (6.5)	5 (20.0)	7 (12.5)
Gastro-intestinal	6 (19.4)	3 (12.0)	9 (16.1)
Respiratory	4 (12.9)	2 (8.0)	6 (10.7)
Ocular	1 (3.2)	1 (4.0)	2 (3.6)
Urogenital	9 (29.0)	3 (12.0)	12 (21.4)
Endocrine	9 (29.0)	3 (12.0)	12 (21.4)
Musculoskeletal	8 (25.8)	7 (28.0)	15 (26.8)
Other	17 (54.8)	10 (40.0)	27 (48.2)
**Allergy/intolerance**^c^*n* (%)			
Yes	9 (29.0)	8 (32.0)	17 (30.4)
No	22 (71.0)	17 (68.0)	39 (69.6)

^a^When the same drug was recorded twice with two different or two of the same doses, this counted as one medication

^b^Number of patients with at least one condition within each category

^c^Number of patients with at least one allergy

After baseline data collection, four patients withdrew after a GP practice withdrew from the study. In addition, there were a further 3 patient withdrawals due to ill health (2 in NI, 1 in the ROI; all from the control arm). Primary outcome data (i.e. GP record data for medication appropriateness assessment) (see ‘Primary outcomes’ section) were available for 47 patients at 9 months (see Table 3). Therefore, 47 of the 68 recruited patients were deemed to be retained in the study, giving an overall retention rate of 69.1%. The CONSORT diagram showing patient recruitment and retention is presented in Fig. 1.

**Table 3:** Treatment after trial entry

	Intervention ***n*** = 38	Control ***n*** = 30
**Patient retention** *n* (%)		
Number with primary outcome (at 6 months)	24 (63.2)	25 (83.3)
Number at end of study with primary outcome (at 9 months)	24 (63.2)	23 (76.7)^a^
Number at end of study with primary or secondary outcomes (at 9 months)	27 (71.1)^b^	22 (73.3)
**Post-randomisation withdrawal of patients ***n* (%)		
Protocol deviation	0 (0.0)	0 (0.0)
Lost to follow-up	0 (0.0)	0 (0.0)
Withdrawal of consent by patient	7 (18.4)	8 (26.7)
Death	0 (0.0)	0 (0.0)
Other (including withdrawal of GP practice)^c^	4 (10.5)	0 (0.0)

^a^Primary outcome data were collected for 1 patient prior to withdrawal

^b^Three patients were followed up for the patient-reported outcome questionnaires from the GP practice which withdrew after baseline data collection

^c^Other reasons: *n* = 4 patients decided to withdraw from the GP practice which withdrew after baseline data collection (intervention arm)

### Primary and secondary outcomes

Six- and 9-month data collection for the primary and secondary outcomes took place from May to July 2021 and August to October 2021, respectively. The proportion of complete data across both primary and secondary outcome measures (i.e. secondary data were available to calculate an EQ-5D-5L utility score and a total burden score from the MRB-QoL) was 93.9% (see Additional file 2).

### Medication appropriateness (primary outcome)

GP record data were available for 56, 49 and 47 patients at baseline, 6 and 9 months, respectively, in order to apply STOPP/START criteria. The proportion of patients with at least one instance of PIP, PIM and PPO at each timepoint is presented in Table 4. At baseline, 27 (87.1%) patients in the intervention arm and 20 (80.0%) in the control arm had at least one instance of PIP. At 6 months, this remained relatively consistent [*n* = 21 (87.5%) patients in the intervention arm and *n* = 20 (80.0%) in the control arm] and again at 9 months [*n* = 21 (87.5%) patients in the intervention arm and *n* = 20 (87.0%) in the control arm]. The two STOPP criteria most frequently applied were A1 (any drug prescribed without an evidence-based clinical indication) and A2 (any drug prescribed beyond the recommended duration, where treatment duration is well defined), while the two START criteria most frequently applied were I1 (seasonal trivalent influenza vaccine annually) and I2 (pneumococcal vaccine at least once after age 65 according to national guidelines).

**Table 4:** The proportion of the patients with potential inappropriate prescribing at baseline, 6, and 9 months

	Intervention	Control	% point difference (95% CI)	ICC^a^
**Potentially inappropriate prescribing (PIP; STOPP and START combined)**				
**Baseline ***n* (%)	*n* = 31 27 (87.1)	*n* = 25 20 (80.0)		
**6 months ***n* (%)	*n* = 24 21 (87.5)	*n* = 25 20 (80.0)	7.5 (−13.0, 28.0)	0.0889
**9 months ***n* (%)	*n* = 24 21 (87.5)	*n* = 23 20 (87.0)	0.5 (−18.6, 19.6)	0.0000
**Potentially inappropriate medications (PIMs; STOPP)**				
**Baseline ***n* (%)	*n* = 31 21 (67.7)	*n* = 25 14 (56.0)		
**6 months ***n* (%)	*n* = 24 14 (58.3)	*n* = 25 15 (60.0)	−1.7 (−29.2, 25.9)	0.0000
**9 months ***n* (%)	*n* = 24 16 (66.7)	*n* = 23 15 (65.2)	1.5 (−25.7, 28.6)	0.0000
**Potential prescribing omissions (PPOs; START)**				
**Baseline ***n* (%)	*n* = 31 20 (64.5)	*n* = 25 16 (64.0)		
**6 months ***n* (%)	*n* = 24 17 (70.8)	*n* = 25 14 (56.0)	14.8 (−11.8, 41.5)	0.1395
**9 months ***n* (%)	*n* = 24 17 (70.8)	*n* = 23 13 (56.5)	14.3 (−12.9, 41.5)	0.0952

*CI* confidence intervals, *ICC* intraclass correlation coefficient. No. (%) presented for patients with at least one instance of PIP, PIMs or PPOs

^a^ICCs from mixed-effects models with site as a random effect

### Health economic analysis

#### Intervention-related resource use and costs

GP time input questionnaires were collected from the four intervention arm GP practices which delivered medication reviews. Data from the online video platform indicated that GPs logged onto the online platform more than once and pressed play on the video multiple times (i.e. GPs may have pressed ‘play’ and ‘pause’ while only watching the video once) (see Additional file 3). The intervention-related resources and costs are presented in Table 5. Overall, it was estimated that the GP resource use and costs associated with the PolyPrime intervention equated to £288.15 per patient.

**Table 5:** General practitioner resource use and costs (£ GBP) associated with the PolyPrime intervention

Resource use	***n***^a^	Mean time input min. (SD)	Cost (£)
**Stage 1: planning and preparation for delivery**			
GP time associated with viewing the online video	-	1.72 (−)	7.27
GP time associated with preparing for medication review 1	11	5.55 (5.75)	23.48
GP time associated with preparing for medication review 2	15	8.2 (5.54)	34.69
**Stage 2: delivery**			
GP time associated with undertaking medication review 1	11	16.60 (7.24)	70.22
GP time associated with carrying out any work post medication review 1	6	3.17 (2.48)	13.41
GP time associated with carrying out any other activity related to medication review 1	2	2.50 (3.54)	10.58
GP time associated with undertaking medication review 2	15	12.69 (3.80)	53.68
GP time associated with carrying out any work post medication review 2	11	6.02 (13.33)	25.46
GP time associated with carrying out any other activity related to medication review 2	3	11.67 (2.89)	49.36
**Total cost per patient**			288.15

^a^*n* number of responses/observations available, medication review 1 patient’s initial medication review, medication review 2 patient’s 6-month follow-up medication review

### Health service use and associated costs (secondary outcome: health service use)

GP record data were available for 56, 49 and 47 patients at baseline, 6 and 9 months, respectively, in order to calculate health service use costs. In addition, patient self-report questionnaires reporting the same data were returned by 67, 47 and 47 patients at the same timepoints, respectively.

Total costs by service type, timepoint, group and data collection methods are presented in Table 6. To allow comparison between the two data collection methods, only patients with complete self-reported and GP record health service use costs were included. Costs were generally very similar between the two methods of data collection methods, with the mean total costs over the study period differing by approximately £20. Total costs were also highly correlated (coefficient = 0.95, where 1.00 is perfect correlation). There was a trend towards higher costs in the control group overall; however, this was largely driven by an outlier who had spent 18 nights in hospital over the study period. When this patient was removed from the analysis, the mean difference in overall costs was −£166.93 via self-report and −£130.96 via GP record. There were no reports of participants having any GP home visits, occupational therapy visits or social work visits over the study period.

**Table 6:** Total health service use costs (£) by time point, group and data collection method

	Patient self-report			GP record		
Service	Intervention (***n*** = 22)	Control (***n*** = 20)	Difference (95% ***CI***)	Intervention (***n*** = 22)	Control (***n*** = 20)	Difference (95% ***CI***)
	Mean (95% ***CI***)	Mean (95% ***CI***)		Mean (95% ***CI***)	Mean (95% ***CI***)	
**6-month follow-up**						
Healthcare professional contact	121.77 (67.07, 176.48)	128.15 (80.18, 176.12)	−6.38 (−77.52, 64.77)	119.18 (87.30, 151.06)	141.30 (65.25, 217.35)	−22.12 (−99.24, 55.01)
Hospital contact	246.90 (−35.19, 529.01)	572.10 (−390.77, 1534.97)	−325.19 (−1256.21, 605.83)	396.32 (165.19, 627.44)	260.50 (45.49, 474.61)	136.27 (−171.23, 443.76)
Total	368.68 (71.86, 665.50)	700.25 (−269.04, 1669.54)	−331.57 (−1272.93, 609.79)	515.50 (287.10, 743.90)	401.35 (153.98, 648.72)	114.15 (−211.52, 439.82)
Total by collection method	526.57 (59.62, 993.53)			461.14 (299.60, 622.68)		
**6- to 9-month follow-up**						
Healthcare professional contact	93.32 (49.13, 137.50)	84.25 (42.98, 125.52)	9.07 (−49.86, 68.00)	91.18 (49.48, 132.88)	100.15 (38.27, 162.04)	−8.97 (−80.02, 62.09)
Hospital contact	70.23 (24.42, 116.03)	772.45 (−261.94, 1806.84)	−702.22 (−1654.53, 250.08)	87.73 (20.45, 155.01)	834.45 (−332.89, 2001.79)	−746.72 (−1822.32, 328.88)
Total	163.55 (91.60, 235.49)	856.70 (−185.64, 1899.04)	−693.15 (−1654.43, 268.12)	178.91 (91.11, 266.71)	934.60 (−254.38, 2123.58)	−755.69 (−1852.67, 341.28)
Total by collection method	493.62 (7.36, 979.88)			538.76 (−14.92, 1092.44)		
Overall total cost	532.23 (237.90, 826.55)	1556.95 (−348.47, 3462.37)	−1024.72 (−2802.38, 752.93)	694.41 (460.40, 928.42)	1335.95 (28.32, 2643.58)	−641.54 (−1867.44, 584.35)
**Overall total by collection method**	1020.19 (129.19, 1911.19)			999.90 (387.23, 1612.58)		

*CI* confidence intervals

The total costs of medication from baseline to 6-month follow-up and from 6- to 9-month follow-up are presented in Table 7. Total medication costs over the study period were higher in the intervention arm.

**Table 7:** Medication costs (£) from baseline to 9 months using GP recorded medication data

Time period	Intervention	Control	Difference (95% ***CI***)
	Mean (95% ***CI***)	Mean (95% ***CI***)	
**Baseline to 6-month follow-up**	728.89 (74.77, 1383.01)	500.60 (49.41. 951.79)	228.29 (−539.67, 996.25)
*nI* = 24			
*nC* = 25			
**6- to 9-month follow-up**	105.66 (75.17, 136.15)	192.28 (44.51, 340.05)	−86.62 (−230.28, 57.04)
*nI* = 24			
*nC* = 23			
**Total**	834.55 (186.26, 1482.84)	696.95 (76.47, 1317.43)	137.60 (−736.34, 1011.53)
*nI* = 24			
*nC* = 23			

*nI, nC* number of patients in intervention (I) and control (C) after withdrawals, *CI* confidence intervals

#### Health outcomes (secondary outcome: HRQoL)

Patient HRQoL questionnaires were returned by 67, 47 and 47 patients at baseline, 6 and 9 months, respectively. Data were available to calculate an EQ-5D-5L utility scores for 64, 47 and 46 patients at baseline, 6 and 9 months, respectively, while data were available to calculate an overall MRB-QoL burden score for 58, 37 and 41 patients for the same time points, respectively. EQ-5D-5L utilities and MRB-QoL total burden scores are presented in Table 8.

**Table 8:** EQ-5D-5L utilities and MRB-QoL total burden score, by time point and group

	Intervention				Control				Difference (95% CI)
	Missing ***n*** (%)	Floor effects ***n*** (%)	Ceiling effects ***n*** (%)	Mean (95% ***CI***)	Missing ***n*** (%)	Floor effects ***n*** (%)	Ceiling effects ***n*** (%)	Mean (95% CI)	
**EQ-5D-5L**									
**Baseline**	2 (5.4)	-	6 (20.0)	0.74 (0.64, 0.83)	1 (3.3)	-	8 (21.6)	0.73 (0.65, 0.82)	0.01 (−0.11, 0.13)
*nI* = 37									
*nC* = 30									
**6 months**	0 (0)	-	4 (15.4)	0.75 (0.67, 0.83)	0 (0)	-	5 (23.8)	0.74 (0.63, 0.84)	0.02 (−0.11, 0.14)
*nI* = 26									
*nC* = 21									
**9 months**	1 (4.0)	-	-	0.72 (0.61, 0.83)	0 (0)	-	-	0.77 (0.68, 0.86)	−0.05 (−0.19, 0.08)
*nI* = 25									
*nC* = 22									
**MRB-QoL**									
**Baseline**	5 (13.5)	-	2 (5.4)	23.79 (18.23, 29.35)	4 (13.3)	-	3 (10.0)	26.52 (18.75, 33.30)	−2.73 (−11.85, 6.39)
*nI* = 37									
*nC* = 30									
**6 months**	3 (11.5)	-	2 (7.7)	21.49 (15.12, 27.87)	7 (33.3)	-	2 (9.5)	24.71 (12.82, 36.61)	−3.22 (−15.02, 8.59)
*nI* = 26									
*nC* = 21									
**9 months**	2 (8.0)	-	2 (8.0)	22.44 (15.24, 29.64)	4 (18.2)	-	4 (18.2)	25.27 (15.40, 35.13)	−2.83 (−14.35, 8.70)
*nI* = 25									
*nC* = 22									

*nI, nC* number of patients in intervention (I) and control (C) after withdrawals, *CI* confidence intervals

HRQoL in patients was generally very good, and no floor effects were observed with either instrument. Ceiling effects were observed in the EQ-5D-5L at both baseline (14/67; 20.9%) and 6 months (9/47; 19.2%) and in the MRB-QoL at baseline (6/67; 7.5%), 6 months (4/47; 8.5%) and 9 months (6/47; 12.8%).

### Process evaluation

#### Intervention fidelity

All four intervention components (i.e. online video, patient recall, weekly meetings and prompts/cues) were delivered, received and/or enacted as intended (see Additional file 3). Four GPs accessed the online platform a median of 4 times (range 3–6) and pressed ‘play’ on the video a median of 8 times (range 2–22) during the intervention delivery phase (i.e. before the initial and/or 6-month follow-up medication reviews) (see Additional file 3). The median number of practice meetings held was 2 (range 1–2), and the median number of prompts delivered to the GPs (per intervention patient) was 2 (range 1–8). Initial medication reviews were delivered between October 2020 and January 2021 (*n* = 24) and 6-month follow-up medication review between May and July 2021 (*n* = 24). Five initial medication reviews were delivered during face-to-face appointments, and 19 were conducted via the telephone. Seven 6-month follow-up medication reviews were delivered during face-to-face appointments, and 17 were conducted via the telephone. In addition, four medication review appointments were audio recorded in NI (initial medication review appointments, *n* = 3; 6-month follow-up medication review appointment, *n* = 1), which confirmed that GPs conducted a structured medication review with the patients as intended. Furthermore, no additional BCTs [14] were used by the GP during the process of the medication review.

#### Intervention acceptability

Feedback interviews were conducted with four GPs and three practice managers from the intervention arm practices. GPs and practice staff thought that overall, the intervention was acceptable.

I think it was, emm, obviously there was a lot of work involved, both for you and for us, and eh a lot of questions which you had to ask… But my overall impression was that it was very professional professionally conducted eh study [GPP24_GP1].

Painless, obviously I don’t know what the results were but I’m sure I can see, you know, I suppose what the aim was so obviously, you know, beneficial, did it cause any upset in the practice, no, it was painless, so yeah, no, it was all straightforward enough [GPP24_Practice Staff1].

In addition, GPs and practice staff reported that they were positive about the study procedures including patient recruitment, the support provided by the research team and their overall involvement in the study (i.e. competing questionnaires, conducting medication reviews).

It [the support provided by the research team] was absolutely the great there wasn’t any point where I felt we were kinda em all at sea or anything you know [GPP22_GP1]

Twenty-one of 24 intervention arm patients returned completed feedback questionnaires (response rate 87.5%). Overall, the patients were positive about their involvement in the PolyPrime study with 73.7% scoring their overall experience as either ‘very good’ or ‘good’ and 80.0% stating that they would recommend being involved in the PolyPrime study to a friend or family member. When patients were asked if they liked or disliked attending the medication review appointments, 81.3% responded ‘strongly like’ or ‘like’. Finally, when respondents were asked what could have improved their overall experience of being involved in the study, patients would have liked longer appointments (*n* = 2), but the majority (*n* = 15; 75.0%) also stated that nothing could be improved, and they were happy with the overall experience (*n* = 4).

#### Mechanisms of action

Four medication review appointments were audio recorded in NI, and data collected were supplemented with the feedback interviews conducted with GPs and practice managers. The following potential mechanisms of action were identified: ‘beliefs about capabilities’ and ‘skills’ (online video, BCT: demonstration of the behaviour), ‘memory, attention and decision processes’ and ‘behavioural regulation’ (weekly meetings, BCT: action planning; prompts by practice staff, BCT: prompts/cues) and ‘beliefs about consequences’ (patient recall, BCT: salience of consequences) (see Additional file 4).

### Progression criteria

Assessment of the a priori progression criteria indicated that two concepts met the ‘Go’ criteria (‘GP practice recruitment’ and ‘completeness of the outcome data’), and three concepts met the ‘Amend’ criteria (‘GP practice retention’, ‘patient recruitment’ and ‘patient retention’) (Table 9).

**Table 9:** Final decisions for each progression criteria (‘Stop’, ‘Amend’, ‘Go’) for the PolyPrime study

Concept	Data source(s)	Progression criteria			Final decision
		Stop (unless there are clear and modifiable contextual or design issues that account for this^a^)	Amend	Go	
GP practice recruitment	Recruitment records held by research nurse(s)	If ≤ 5 GP practices are recruited within 8 months	If 6–9 GP practices are recruited and/or it takes longer than predicted (6–8 months)	If ≥ 10 GP practices are recruited to take part in ≤ 6 months	**Go:** 12 GP practices were recruited within 6 months
GP practice retention	Retention records held by research nurse(s)	If ≤ 5 GP practices are retained for the required period	If 6–9 GP practices can be retained for the required period	If ≥ 10 GP practices can be retained for the required period	**Amend:** 9 GP practices were retained until the end of the study period
Patient recruitment	Recruitment records held by Research Fellow/Assistant	If ≤ 59 patients are recruited within 5 months^b^	If 60–95 patients are recruited within 5 months^b^	If ≥ 96 patients are recruited within 5 months^b^	**Amend:** 68 patients were recruited within 4 months^c^
Patient retention	Retention records held by Research Fellow/Assistant	If ≤ 49% of patients are retained for the required period	If 50–79% of patients are retained for the required period	If ≥ 80% of patients are retained for the required period	**Amend:** 69.1% (47 of 68) of patients were retained in the study (i.e. had GP record data available for primary outcome analysis)
Completeness of outcome data	Data collected during the study (CRFs, questionnaires)	If ≤ 49% of each patient self-repor, and GP-reported outcome measure is complete	If 50–79% of each patient self-report and GP-reported outcome measure is complete	If ≥ 80% of each patient self-report and GP-reported outcome measure is complete	**Go:** 93.9% of primary and secondary outcome data were complete (see Additional file 2)

^a^This includes aspects of study and/or data collection procedures that may be modified in advance of a full-scale definitive cRCT

^b^Note that if ethics amendments are made to study recruitment procedures during this time period, the 5-month recruitment period may be extended (up to 8 months) to enable sufficient time to assess patient recruitment rates

^c^Patient recruitment underway from the end of January 2020 to mid-March 2020; paused from mid-March to mid-September 2020; recruitment underway again from mid-September 2020 to mid-November 2020, i.e. approximately 4 months. Recruitment was stopped in mid-November 2020 to allow for sufficient time to undertake follow-up

As one or more of the concepts met the ‘Amend’ criteria, the results of the progression criteria were presented to the TSC. It was agreed that in light of the impact of COVID-19, the concept relating to ‘GP practice retention’ would have met the ‘Go’ criteria. However, it was agreed that there were insufficient data to ascertain if the ‘Go’ criteria for ‘patient recruitment’ and ‘patient retention’ would have been met. The TSC also suggested that further consideration was warranted due to the change in mode of delivering the medication reviews (face-to-face versus telephone and online/video consultations) and developments to the primary care workforce (i.e. introduction of practice-based pharmacists).

### Serious adverse events

Over the intervention delivery phase, no SAEs were reported by either the control or intervention arm GP practices.

### Sample size

Four sample size calculation scenarios were developed as shown in Table 10. These were based on a 10% or 15% reduction in PIP (starting with a baseline figure of 85% of patients with PIP), a cluster size of 20, an ICC of either 0.01 or 0.025 (as reported in the OPTI-SCRIPT study [35]) and 20% loss to follow-up, with 90% power and a statistical significance of 5% (two sided), between the randomised groups. A 10% or 15% reduction in PIP was selected as this represented a clinically significant reduction in the proportion of patients with PIP [35].

**Table 10:** Sample size calculation scenarios

Reduction in PIP (%)	Cluster size	ICC	Loss to follow-up (%)	Total sample size	No. of clusters
10	20	0.01	20	997	50
10	20	0.025	20	1236	62
15	20	0.01	20	479	24
15	20	0.025	20	594	30

*ICC* intraclass correlation coefficient, *PIP* potentially inappropriate prescribing

## Discussion

The current study builds on the existing evidence base by further testing a theory-based intervention, originally developed in NI, in a larger cross-border setting. The external pilot cRCT primarily aimed to assess the feasibility of delivering the PolyPrime intervention [13] and was not intended to provide definitive results in terms of the effectiveness or cost-effectiveness of the intervention package. The feasibility of recruitment and study procedures, including collecting data on medication appropriateness (from GP records), quality of life and health service use (i.e. hospitalisations), was also explored. This research will advance the existing literature by helping to address the uncertainties identified in the previous small feasibility study [11] surrounding sampling and recruitment, intervention delivery and outcome data collection procedures across the two healthcare jurisdictions.

### Recruitment and retention

The GP practice recruitment strategies (expression of interest letters followed by telephone calls by research nurses) were successful with 12 GP practices recruited within 6 months. Three GP practices withdrew from the study which met the ‘Amend’ criteria. All three withdrawals were due to COVID-related issues, and as such, no modifications to improve GP recruitment are deemed necessary for a future trial. It is important to consider the impact COVID-19 has had on primary care [36] and to conducting research in this setting [37]. Strategies to enhance practice recruitment and retention and reduce the burden of participating in a future trial may be warranted. We acknowledge that the overall practice recruitment rate was low (i.e. 12/201 practices; 6%). However, the funding programme that supported the PolyPrime study was also supporting a number of other primary care-based studies that were also seeking to recruit general practices at approximately the same time. This is borne out by comparing the participation rate for the PolyPrime study (6%) and another medicines management study in general practice in which the participation rate for practices was 5.3% (Murphy, personal communication).

Patient recruitment was well underway pre-COVID-19; however, when the study restarted, further patient recruitment efforts were not possible in all practices due to the ongoing COVID restrictions in place, access to practices and research nurses’ availability. However, it is uncertain if the patient recruitment strategy would have led to the minimum recruitment target being achieved (*n* = 96) to meet the ‘Go’ criteria. Patient retention was also largely impacted by COVID-19; several patients withdrew after the study restarted and when a practice withdrew. Although only three patients withdrew due to health reasons, close attention to patient retention strategies should be considered.

### Data collection

This pilot study has shown that it was feasible to collect GP record and patient self-reported data, with high return rates and completeness of data. In addition, it was feasible to collect the GP record data required to apply the STOPP/START criteria in order to assess medication appropriateness. Although time-consuming in terms of collecting the required data from GP records and conducting the assessment of medication appropriateness using the STOPP/START criteria [16], medication appropriateness should be selected as the primary outcome in a future trial. This aligns with a core outcome set for trials aimed at improving appropriate polypharmacy in older people in primary care [38] developed by members of the research team, in which medication appropriateness was one of the seven highest ranked outcomes.

The CRF also included a health service use section, which, along with a patient self-reported version, were comparable in terms of the overall costs yielded, suggesting that either method could be used in a future trial as part of the cost-effectiveness analysis. The choice of which method (GP record versus patient self-report) to use may be driven by whether the burden of the data collection should lie with the patient or the research nurses. The piloting of these CRFs has found that some services were not used, or rarely used, by patients, and this will facilitate the design of a more streamlined data collection forms in the future. It must be noted however that patients’ access to health care may have been disrupted due to the pandemic leading to inflated contacts in some services (e.g. telephone calls to GP rather than face-to-face contact) and reduced contacts in other services (e.g. hospital outpatient visits) [39]. The nature of these contacts may also have changed (e.g. from face-to-face to online/telephone consultations [36]) which may have led to under-reporting due to the questionnaire and CRFs not being designed to capture these subtle nuances.

The study has also shown that patient self-reported HRQoL data can be collected to a high degree of completeness. Higher levels of missing data were observed in the MRB-QoL, which was not unexpected since 31 items are required to be completed to calculate a total MRB-QoL [28] compared to just 5 items in the EQ-5D-5L [27]. Overall, while there are some aspects of commonality between the two HRQoL instruments, both should be incorporated into a future RCT to ensure the full impact of medication reviews is measured. Using a selection of the subscales in the MRB-QoL may improve levels of completeness.

While the COVID-19 pandemic impacted on the collection of baseline health service use data, this had little impact on the assessment of the feasibility of a future cost-effectiveness analysis. The resources used in the set-up and delivery of the intervention mainly related to GP time spent planning (i.e. watching the online video) and delivering the medication review appointments, estimated at £288.15 per patient. However, as GPs accessed the online platform more than once and pressed ‘play’ on the video multiple times, GP time associated with viewing the online video may be underestimated. If the intervention was adopted into clinical practice, this input is unlikely to be required regularly, perhaps only as part of annual Continuing Professional Development.

### Process evaluation

The PolyPrime intervention components were successfully implemented in the two healthcare systems and was deemed to be acceptable to GPs, practice staff and patients. When the PolyPrime study restarted in July 2020, the mode in which GPs could deliver the medication review was adapted because of COVID-19 restrictions being in place. While several face-to-face medication reviews still took place, the majority were conducted via telephone; this did not impact on the quality of the medication reviews delivered, based on the audio recordings of a small number of medication reviews. Indeed, over the course of the COVID-19 pandemic, there have been significant changes to primary care services in NI and the ROI, including an increase in both telephone and video consultations [36]. It was also evident that weekly meetings took place, and prompts were delivered to the GPs, both of which were deemed useful by GPs and practice staff. In addition, potential mechanisms of action have been identified which mirror the original Theoretical Domains Framework (TDF) domains [40] used in the underpinning intervention development work [9, 10].

As noted in the ‘Results’, we did not identify additional BCTs in our process evaluation beyond those specified a priori; however, it is possible that additional BCTs may have been activated by some of the intervention content. While evidence suggests that post hoc coding of BCTs in interventions is generally reliable, this is more difficult for the kind of commonly applied BCTs that we used in the PolyPrime intervention [40]. Future research could undertake a detailed BCT content analysis of some of the new intervention content, e.g. use of the NO TEARS checklist and the NICE guidelines, to ascertain whether use of these materials in complex interventions potentially activates additional BCTs and related mechanisms of action.

### Strengths and limitations

The PolyPrime study is based on a systematic, theory-based approach using the UK Medical Research Council’s complex intervention framework [14, 41–43]. The a priori progression criteria along with oversight from the TSC contributed to an objective assessment on whether to proceed to a future definitive cRCT. The study also benefitted from being multidisciplinary in nature, coupled with the involvement of patient representatives in terms of the development of rigorous data collection tools. The pilot study was impacted by the COVID-19 pandemic which led to the study being suspended for a number of months in 2020. This suspension had a direct impact on GP practice retention as well as both patient recruitment and retention, which has been reported across clinical trials [37, 44]. While the study was successfully conducted in two healthcare systems, which included both small and larger GP practices, it was limited by the small patient sample size achieved. Therefore, the outcome data presented should be interpreted with caution.

Three aspects of progression relating to GP practice retention, patient recruitment and patient retention met the ‘Amend’ threshold as opposed to the ‘Go’ progression criteria. It will be important to consider the effectiveness of study procedures and strategies to increase recruitment and retention rates in a future trial. A future definitive cRCT may require an internal pilot to confirm the effectiveness of patient recruitment strategies, the ability to retain patients for the duration for the study and the changes made to the mode of intervention delivery (i.e. the use of telephone and video consultations). It is also important to highlight that the PolyPrime study was developed based on intervention development and feasibility work conducted in 2014 and 2015 [9–11]. The primary care workforce has changed significantly since then with the introduction of practice-based pharmacists in NI. As such, further work is also needed to investigate potential adaptations to the delivery of the intervention package in terms of the workforce in primary care (i.e. the role of practice-based pharmacists (PBPs)). A series of sample size calculations were undertaken, and the number of clusters and total sample sizes in each scenario will need careful consideration in any planning for a future definitive trial.

## Conclusions

Despite challenges faced due to the COVID-19 pandemic, this study has demonstrated that it may be feasible to conduct a theory-based intervention aimed at improving appropriate polypharmacy in older people in primary care across two healthcare jurisdictions. The results support the future testing of the PolyPrime intervention in a definitive trial; however, uncertainties remain surrounding patient recruitment and retention. A future definitive cRCT may also need to further explore how the PolyPrime study could be adapted to take into consideration the recent changes in primary care including the mode of delivering medication reviews and the role which PBPs play in their delivery.

## Supplementary Information

**Additional file 1: Supplementary Table 1.** CONSORT checklist.

**Additional file 2: Supplementary Table 2.** Completeness of outcome data.

**Additional file 3: Supplementary Table 3.** Delivery of the intervention components.

**Additional file 4: Supplementary Table 4.** Potential mechanisms of action in the PolyPrime intervention.

## Data Availability

The datasets used and/or analysed during the current study are available from the corresponding author on reasonable request.

## Abbreviations

BCT: Behaviour change technique
CI: Confidence intervals
CONSORT: Consolidated Standards of Reporting Trials
COPD: Chronic obstructive pulmonary disease
cRCT: Cluster randomised controlled trial
CRF: Care report form
EQ-5D-5L: EuroQol-5 dimension-5 level questionnaire
GMS: General medical services
GP: General practitioner
GPP: General practitioner practice
HSE: Health Service Executive
HRQoL: Health-related quality of life
MRB-QoL: Medication-related burden quality of life
NHS: National Health Service
NI: Northern Ireland
NICE: National Institute for Health and Care Excellence
NICRN: Northern Ireland Clinical Research Network
NO TEARS: Need and indication, Open questions, Tests and monitoring, Evidence and guidelines, Adverse events, Risk reduction or prevention, Simplification and switches
PBP: Practice-based pharmacist
PIM: Potentially inappropriate medication
PIP: Potentially inappropriate prescribing
PPI: Patient and public involvement
PPO: Potential prescribing omission
RCT: Randomised controlled trial
REC: Research Ethics Committee
ROI: Republic of Ireland
SAE: Serious adverse event
SD: Standard deviation
START: Screening Tool to Alert doctors to Right Treatment
STOPP: Screening Tool of Older People’s potentially inappropriate Prescriptions
TCD: Trinity College Dublin
TDF: Theoretical Domains Framework
TFA: Theoretical Framework of Acceptability
TSC: Trial Steering Committee
WHO: World Health Organisation
UK: United Kingdom

## References

[1] Cadogan CA, Ryan C, Hughes CM (2016). Appropriate polypharmacy and medicine safety: when many is not too many. Drug Saf.

[2] Guthrie B, Makubate B, Hernandez-Santiago V, Dreischulte T (2015). The rising tide of polypharmacy and drug-drug interactions: population database analysis 1995–2010. BMC Med.

[3] McGarrigle C, Donoghue O, Scarlett S, Kenny RA. Health and wellbeing: active ageing for older adults in Ireland. Dublin: The Irish Longitudinal Study on Ageing (TILDA): 2017. https://tilda.tcd.ie/publications/reports/pdf/w3-key-findings-report/TILDA%20Wave%203%20Key%20Findings%20report.pdf. Accessed 26 Feb 2022.

[4] Donaldson LJ, Kelley ET, Dhingra-Kumar N, Kieny MP, Sheikh A (2017). Medication without harm: WHO’s third global patient safety challenge. Lancet..

[5] World Health Organisation (2019). Medication safety in polypharmacy.

[6] Duncan EM, Francis JJ, Johnston M, Davey P, Maxwell S, McKay GA (2012). Learning curves, taking instructions, and patient safety: using a theoretical domains framework in an interview study to investigate prescribing errors among trainee doctors. Implement Sci.

[7] Mucklow J, Bollington L, Maxwell S (2012). Assessing prescribing competence. Br J Clin Pharmacol.

[8] Rankin A, Cadogan C, Ryan C, Clyne B, Smith SM, Hughes C (2018). Interventions to improve the appropriate use of polypharmacy for older people. Cochrane Database Syst Rev.

[9] Cadogan CA, Ryan C, Francis JJ, Gormley GJ, Passmore P, Kerse N (2015). Improving appropriate polypharmacy for older people in primary care: selecting components of an evidence-based intervention to target prescribing and dispensing. Implement Sci.

[10] Cadogan CA, Ryan C, Francis JJ, Gormley GJ, Passmore P, Kerse N (2016). Development of an intervention to improve appropriate polypharmacy in older people in primary care using a theory-based method. BMC Health Serv Res.

[11] Cadogan CA, Ryan C, Gormley GJ, Francis JJ, Passmore P, Kerse N (2017). A feasibility study of a theory-based intervention to improve appropriate polypharmacy for older people in primary care. Pilot Feasibility Stud.

[12] Gorman A, Rankin A, Barry H, Cadogan C, Gormley G, Fahey T (2020). A qualitative study to refine a theory-based intervention to improve appropriate polypharmacy in older people in primary care. Int J Pharm Pract.

[13] Rankin A, Cadogan CA, Barry HE, Gardner E, Agus A, Molloy GJ (2021). An external pilot cluster randomised controlled trial of a theory-based intervention to improve appropriate polypharmacy in older people in primary care (PolyPrime): study protocol. Pilot Feasibility Stud.

[14] Michie S, Richardson M, Johnston M, Abraham C, Francis J, Hardeman W (2013). The behavior change technique taxonomy (v1) of 93 hierarchically clustered techniques: building an international consensus for the reporting of behavior change interventions. Ann Behav Med.

[15] Lewis T (2004). Using the NO TEARS tool for medication review. BMJ..

[16] O’Mahony D, O’Sullivan D, Byrne S, O’Connor MN, Ryan C, Gallagher P (2015). STOPP/START criteria for potentially inappropriate prescribing in older people: version 2. Age Ageing.

[17] National Institute for Health and Care Excellence. Medicines optimisation: the safe and effective use of medicines to enable the best possible outcomes [NG5]. 2015. www.nice.org.uk/guidance/ng5. Accessed 18 Feb 2022. 26180890

[18] Health Service Executive (HSE). Chronic Disease Management Programme: 2020 https://www.hse.ie/eng/about/who/gmscontracts/2019agreement/chronic-disease-management-programme/circular-chronic-disease-management-nco-04-2020.pdf. Accessed 28 Feb 2022.

[19] Rankin A, Molloy GJ, Cadogan CA, Barry HE, Gorman A, Ryan C (2021). Protocol for a process evaluation of an external pilot cluster randomised controlled trial of a theory-based intervention to improve appropriate polypharmacy in older people in primary care: the PolyPrime study. Trials..

[20] Sergeant ESG. Epitools epidemiological calculators, Ausvet: 2018. Available at: http://epitools.ausvet.com.au. Accessed 1 Mar 2022.

[21] Curtis L, Burns A. Unit Costs of Health and Social Care 2020. Personal Social Services Research Unit, University of Kent, Canterbury: 2020. https://www.pssru.ac.uk/project-pages/unit-costs/unit-costs-2020/. Accessed 25 Feb 2022.

[22] Department of Health. Reference Costs 2019-20, Department of Health: 2020.

[23] Smith S, Jiang J, Normand C, O’Neill C (2021). Unit costs for non-acute care in Ireland 2016-2019 [version 1; peer review: 1 approved]. HRB Open Res.

[24] Healthcare Pricing Office. ABF Admitted Patient Price List 2019, Healthcare Pricing Office: 2019. https://www.hpo.ie/abf/ABF2019AdmittedPatientPriceList.pdf. Accessed 25 Feb 2022.

[25] Organisation for Economic Cooperation and Development (OECD). Purchasing power parities (PPP) (indicator): 2022. 10.1787/1290ee5a-en. Accessed 25 Feb 2022.

[26] Heslin M, Babalola O, Ibrahim F, Stringer D, Scott D, Patel A (2018). A comparison of different approaches for costing medication use in an economic evaluation. Value Health.

[27] Herdman M, Gudex C, Lloyd A, Janssen MF, Kind P, Parkin D (2011). Development and preliminary testing of the new five-level version of EQ-5D (EQ-5D-5L). Qual Life Res.

[28] Mohammed MA, Moles RJ, Hilmer SN, O’Donnel LK, Chen TF (2018). Development and validation of an instrument for measuring the burden of medicine on functioning and well-being: the medication-related burden quality of life (MRB-QoL) tool. BMJ Open.

[29] van Hout B, Janssen MF, Feng YS, Kohlmann T, Busschbach J, Golicki D (2012). Interim scoring for the EQ-5D-5L: mapping the EQ-5D-5L to EQ-5D-3L value sets. Value Health.

[30] National Institute for Health and Care Excellence. Guide to the methods of technology appraisal [PMG9]. 2013. https://www.nice.org.uk/process/pmg9/resources/guide-to-the-methods-of-technology-appraisal-2013-pdf-2007975843781. Accessed 25 Feb 2022. 27905712

[31] Sekhon M, Cartwright M, Francis JJ (2017). Acceptability of healthcare interventions: an overview of reviews and development of a theoretical framework. BMC Health Serv Res.

[32] Carey RN, Connell LE, Johnston M, Rothman AJ, de Bruin M, Kelly MP (2019). Behavior change techniques and their mechanisms of action: a synthesis of links described in published intervention literature. Ann Behav Med.

[33] Borrelli B, Sepinwall D, Ernst D, Bellg AJ, Czajkowski S, Breger R (2005). A new tool to assess treatment fidelity and evaluation of treatment fidelity across 10 years of health behavior research. J Consult Clin Psychol.

[34] Lancaster GA, Thabane L (2019). Guidelines for reporting non-randomised pilot and feasibility studies. Pilot Feasibility Stud.

[35] Clyne B, Smith SM, Hughes CM, Boland F, Bradley MC, Cooper JA (2015). Effectiveness of a multifaceted intervention for potentially inappropriate prescribing in older patients in primary care: a cluster-randomized controlled trial (OPTI-SCRIPT study). Ann Fam Med.

[36] Thornton J (2020). Covid-19: how coronavirus will change the face of general practice forever. BMJ..

[37] Shiely F, Foley J, Stone A, Cobbe E, Browne S, Murphy E, Kelsey M, Walsh-Crowley J, Eustace JA (2021). Managing clinical trials during COVID-19: experience from a clinical research facility. Trials..

[38] Rankin A, Cadogan CA, Ryan C, Clyne B, Smith SM, Hughes CM (2018). Core outcome set for trials aimed at improving the appropriateness of polypharmacy in older people in primary care. JAGS..

[39] Fraser C, Fisher R. How has the COVID-19 pandemic impacted primary care? Health Foundation. 2021. https://www.health.org.uk/news-and-comment/charts-and-infographics/how-has-the-covid-19-pandemic-impacted-primary-care. Accessed 28 Mar 2022.

[40] Abraham C, Wood CE, Johnston M, Francis J, Hardeman W, Richardson M, Michie S (2015). Reliability of identification of behavior change techniques in intervention descriptions. Ann Behav Med.

[41] Cane J, O’Connor D, Michie S (2012). Validation of the theoretical domains framework for use in behaviour change and implementation research. Implement Sci.

[42] Craig P, Dieppe P, Macintyre S, Michie S, Nazareth I, Petticrew M (2008). Developing and evaluating complex interventions: the new Medical Research Council guidance. BMJ..

[43] Medical Research Council. Developing and evaluating complex interventions: new guidance: 2008. www.mrc.ac.uk/documents/pdf/complex-interventions-guidance/. Accessed 24 Feb 2022.

[44] Thornton J (2020). Clinical trials suspended in UK to prioritise covid-19 studies and free up staff. BMJ..

