# Supplementary material for: An external pilot cluster randomised controlled trial of a theory-based intervention to improve appropriate polypharmacy in older people in primary care (PolyPrime)
Source: Pilot Feasibility Stud. 2022 Sep 10;8:203. doi: 10.1186/s40814-022-01161-6 (PMC9463515; doi:10.1186/s40814-022-01161-6)
Supplement: Supplementary file 2 — Additional file 2: Supplementary Table 2. Completeness of outcome data. [file 40814_2022_1161_MOESM2_ESM.docx]

| **Supplementary Table 2** Completeness of primary and secondary outcome measures in the PolyPrime study | | |
| --- | --- | --- |
| **Data source** | **Patients with available data**  **(n)** | **Completeness of outcome measure (%)^a^** |
| **Primary outcome** | | |
| **Medication appropriateness (GP-record)** | | |
| Baseline | 56 | 100 |
| 6-month | 49 | 100 |
| 9-month | 47 | 100 |
| **Secondary outcomes** | | |
| **EQ-5D-5L** | | |
| Baseline | 64 | 94.1 |
| 6-month | 47 | 92.2 |
| 9-month | 46 | 93.9 |
| **MRB-QoL** |  |  |
| Baseline | 58 | 85.3 |
| 6-month | 37 | 72.5 |
| 9-month | 41 | 83.7 |
| **Health service use (Patient-reported)** |  |  |
| Baseline | 67 | 98.5 |
| 6-month | 47 | 92.2 |
| 9-month | 47 | 95.9 |
| **Health service use (GP-record)** |  |  |
| Baseline | 56 | 100 |
| 6-month | 49 | 100 |
| 9-month | 47 | 100 |
| **Overall** | - | **93.9** |
| MRB-QoL=medication-related quality of life  ^a^ Completeness of ED-5D-5L and MRB-QoL based on whether data was available to calculate an EQ-5D-5L utility score and a MRB-QoL total burden score | | |
