# Supplementary material for: An external pilot cluster randomised controlled trial of a theory-based intervention to improve appropriate polypharmacy in older people in primary care (PolyPrime)
Source: Pilot Feasibility Stud. 2022 Sep 10;8:203. doi: 10.1186/s40814-022-01161-6 (PMC9463515; doi:10.1186/s40814-022-01161-6)
Supplement: Supplementary file 3 — Additional file 3: Supplementary Table 3. Delivery of the intervention components. [file 40814_2022_1161_MOESM3_ESM.docx]

| **Supplementary Table 3** Delivery of the intervention components | | | |
| --- | --- | --- | --- |
| **Data collected** | **Total** | **NI (n=2)** | **ROI**  **(n=2)** |
| Median number of times the GPs accessed the video (i.e. logged into the online platform) (range) | 4 (3-6) | 3 (3-3) | 5 (3-6) |
| Median number of times video was played (range) | 8 (2-22) | 8 (7-8) | 15 (7-22) |
| Median number of weekly meetings held (range) | 2 (1-2) | 2 (1-2) | 2 (1-2) |
| Number of initial medication review appointments scheduled (n) | 25^a^ | 15^a^ | 10 |
| Number of initial medication review appointments attended (n) | 24 | 14 | 10 |
| Number of six-month follow-up medication review appointments scheduled (n) | 24 | 14 | 10 |
| Number of six-month follow-up medication review appointments attended (n) | 24 | 14 | 10 |
| Median number of prompts delivered (range)^b^ | 2 (1-8) | 2 (2-8) | 1 (0-2) |
| *Verbal prompts* | 1 (0-2) | 1 (1-4) | 2 (1-2) |
| *Electronic prompts* | 1 (0-4) | 1 (1-4) | 0 |
| Median number of prompts delivered before initial medication reviews (range)^b^ | 2 (1-2) | 2 (2-2) | 2 (1-2) |
| *Verbal prompts* | 1 (1-2) | 1 (1-1) | 2 (1-2) |
| *Electronic prompts* | 1 (0-1) | 1 (1-1) | 0 |
| Median number of prompts delivered before six-month follow-up medication reviews (range)^b^ | 4 (1-8) | 4 (1-4) | 2 (1-2) |
| *Verbal prompts* | 2 (1-4) | 2 (1-4) | 0 |
| *Electronic prompts* | 2 (0-4) | 2 (1-4) | 2 (1-2) |
| NI Northern Ireland, ROI Republic of Ireland  ^a^ One initial medication review cancelled and re-scheduled  ^b^ Based on data collected from three intervention arm GP practices | | | |
