# Supplementary material for: An external pilot cluster randomised controlled trial of a theory-based intervention to improve appropriate polypharmacy in older people in primary care (PolyPrime)
Source: Pilot Feasibility Stud. 2022 Sep 10;8:203. doi: 10.1186/s40814-022-01161-6 (PMC9463515; doi:10.1186/s40814-022-01161-6)
Supplement: Supplementary file 4 — Additional file 4: Supplementary Table 4. Potential mechanisms of action in the PolyPrime intervention. [file 40814_2022_1161_MOESM4_ESM.docx]

| **Supplementary Table 4** Potential mechanisms of action (MoA) in the PolyPrime intervention | | | |
| --- | --- | --- | --- |
| **Intervention component** | **Impact** | **MoA** | **Illustrative quotes** |
| Online video | Changes in the GPs’ beliefs about capabilities (e.g. their self-efficacy) to prescribe appropriate polypharmacy during a consultation with an older patient | GPs’ beliefs about capabilities  Skills | *“Yeah, happy enough… I think so, think so yeah”* [GPP11_GP1].  *“Em pretty confident, I think like most, most of the actual kind communication skills, clinical acumen that is demonstrated in the video I think is something that most GPs would be pretty self-confident about. Em so again, I think it’s just having the em, the opportunity and the time and whether that’s time to do it opportunistically or making the time as part of routine healthchecks. Em, but that’s, that probably would be the main thing that, just, not being focused on and being able to do it at the appropriate time”* [GPP22_GP1].  *“I would say I was very confident, increasingly confident, ehh I would have been confident to conduct such an interview in any case, but the eh the the video was of enhanced system”* [GPP24_GP1]. |
| Weekly meetings | Weekly meetings are perceived to be effective in GPs making plans to schedule patient appointments | Memory, attention and decision processes  Behavioural regulation | *“I thought it was useful and ehh it wasn’t at all disruptive I mean it was one, was one meeting per week”* [GPP24_GP1].  *“Yeah I think what we did was we had a chat with* [practice manager] *just to eh see and it was really kind of I suppose more the logistics of it sort of fitting patients in around people’s annual leave and there was quite a bit of sickness as well in the practice so just trying to manoeuvre when you could get the free time to do it em but yeah as a process absolutely sound no problems yeah”* [GPP13_GP1]. |
| Prompts by practice staff | Prompts from practice staff were perceived to be effective in reminding GPs to deliver a medication review to patients | Memory, attention and decision processes  Behavioural regulation | *“Electronic, it was put on the computer system, once we’d agreed a plan when we were going to do it because I think on our I’m talking about our second review it was I was I had come back from holidays so that was all set up for me electronically on the computer system… Em I think it was because we had I think that just worked better at the time particularly so we’ve adopted cause our system’s changed so much because of Covid… everything is just done on the day so that had to be added on so that had to be added on as extras so em yes that was important cause it wasn’t in our system for booked appointments”* [GPP11_GP1].  *“Em the verbal is okay I do like the electronic prompt… em because em there’s so much going on you know I’ll say something to them and it’ll go out of their head I prefer to do em an electronic prompt or I’ve even put a note in their eh in tray their you know their actual in tray em to say this is doing but mainly em I would have maybe emailed* [GP] *directly and sort of said to him you know ‘this is what we’re doing we’ve agreed these dates’ em and then we would have put them I would have put them on to the screen I would have marked them exactly what they were so he knew when he if he’d forgotten that when he came that morning”* [GP13_PM1]. |
| Patient recall | GPs reporting positive attitudes towards the medication review | Beliefs about consequences | *“Em enjoyable* [conducting the medication reviews] *em and I think nice to have like you know if it was part of my normal day I’d be quite happy compared again compared to some of the consultations you do with people no em grand”* [GPP11_GP1].  *“Eh well I think it certainly was a very valuable exercise* [medication reviews] *because polypharmacy is eh is quite a problem in in eh medical practice particularly in elderly patients… so i think it was a very very valid worthy eh intervention”* [GPP24_GP1]. |
